# Laugh

**Published:** 1893-09

**Authors:** 


LAUGH.—Learn to laugh. A good laugh is better than medicine. Learn how to tell a story. A well-told story is as welcome as a sunbeam in a sick-room. Learn to keep your own troubles to yourself. The world is too busy to care for your ills and sorrows. Learn to stop croaking. If you cannot see any good in the world, keep the bad to yourself. Learn to hide your pains and aches under a pleasant smile. No one cares to hear whether you have the earache, headache or rheumatism. Don't cry. Tears do well enough in novels, but they are out of place in real life.

